# Functional Nausea in Children: A Review of the Literature and Need for Diagnostic Criteria

**DOI:** 10.3390/children3010005

**Published:** 2016-03-10

**Authors:** Alexandra C. Russell, Amanda L. Stone, Lynn S. Walker

**Affiliations:** 1 Department of Pediatrics, Vanderbilt University School of Medicine, Nashville, TN 37232, USA; Lynn.Walker@Vanderbilt.edu; 2 Department of Psychology and Human Development, Vanderbilt University, Nashville, TN 37203, USA; Amanda.Sherman@Vanderbilt.edu

**Keywords:** functional nausea, functional gastrointestinal disorders, chronic idiopathic nausea, autonomic dysfunction, integrative medicine

## Abstract

Nausea is common amongst children with functional gastrointestinal disorders and is associated with a high burden of somatic and psychosocial comorbidities in both the short and long-term. Current treatments including medications, phytotherapy, stress-reduction techniques, and gastric electrical stimulation for recalcitrant cases, are reviewed. Functional nausea merits its own diagnostic criteria as a pediatric functional gastrointestinal disorder.

## 1. Introduction

Nausea is a complex and debilitating symptom that frequently affects children with functional abdominal pain (FAP); half of children with functional abdominal pain experience nausea on a regular basis [1,2,3]. Given the high prevalence of functional abdominal pain among children and adolescents and the pervasiveness of somatic symptoms in this population [4], the burden of pediatric functional nausea is likely substantial. Functional nausea remains relatively unexamined in the pediatric literature. Much of this stems from the lack of pediatric diagnostic criteria such as those provided by the current Rome III criteria for other functional gastrointestinal disorders (FGIDs). In adults, chronic idiopathic nausea (CIN) is defined as a functional gastroduodenal disorder with the following clinical criteria needed for diagnosis: bothersome nausea occurring at least several times per week, usually not associated with emesis, and in the absence of endoscopic abnormalities or metabolic disease [5]. Criteria must be met for the prior three months, with symptom onset at least six months before diagnosis. Many children who meet the adult criteria for CIN also meet criteria for other FGIDs [1], illustrating the need for pediatric specific characterization and classification [6].

This patient group can be very challenging to treat. There are presently no known effective interventions for pediatric functional nausea, making it difficult to alleviate suffering in these children [6]. The lack of evidence-based interventions adds distress for both physicians and patients and likely exacerbates long-term suffering and comorbid disability or psychological disorders in these patients.

## 2. Proposed Pathophysiology of Functional Nausea

The etiology of functional nausea is poorly described and multifactorial, likely involving the autonomic nervous system, biologic and mechanical triggers, psychosocial factors, and dietary and microbial effects. Nausea is a subjective feeling of the need to vomit, experienced as an unpleasant but painless epigastric sensation and typically accompanied by signs of autonomic arousal such as pallor, sweating, increased salivation, and tachycardia [7]. It can be triggered by chemical, visceral, central nervous system, and vestibular stimulation, as well as by strong memories or emotions [8].

### 2.1. Biophysical Etiology

Nausea can be viscerally triggered by gastric irritants, physical and psychological stressor-induced gastric acid secretion, and by gastric distention [8]. These pathways are regulated by the vagus and sympathetic nerves, which transmit to the nucleus tractus solitarius in the brainstem and the nodosum ganglion to initiate the emetic reflex [9]. These pathways involving the sympathetic branch of the autonomic nervous system may explain recent work linking autonomic disturbance with pediatric functional nausea. Individuals susceptible to nausea have electrogastrographic and autonomic changes consistent with heightened sympathetic nervous system activity and reduced parasympathetic activity not seen in those who do not experience nausea [10]. Children with chronic nausea often manifest signs of dysautonomia, including decreased heart rate variability [11], orthostatic intolerance [11,12], and gastric dysrhythmias [12,13]. Other FGIDs, such as functional abdominal pain and cyclic vomiting syndrome, have shown similar associations with autonomic dysfunction [14,15]. Alterations in autonomic nervous system functioning may also explain some of the co-morbidities reported with chronic nausea; it has been described in patients with both childhood anxiety and chronic nausea [11,16,17]. The pathophysiology of functional nausea, however, cannot be attributable to dysautonomia alone, as clinical presentation is not predictive of postural tachycardia syndrome [18]. Similarly, although nausea is a cardinal feature of gastroparesis, gastric emptying abnormalities do not appear to account for most cases of functional nausea [19,20].

It has been suggested that chronic functional nausea may be more on the continuum of gastroparesis than that of dysautonomia or FGIDs [21]. High-resolution electrical mapping and electron microscopy of full-thickness gastric biopsies have shown that adults with chronic unexplained nausea and vomiting have pathologic cellular and bioelectrical abnormalities similar to individuals with gastroparesis [21]. This may be secondary to fewer interstitial cells of Cajal, the cells that generate and propagate gastric slow waves, in those with chronic nausea [21]. While electromechanical dysfunction has been investigated for pediatric functional dyspepsia [22], it has not yet been assessed in pediatric functional nausea.

### 2.2. Psychosocial Etiology

Given the high incidence of stress, anxiety, and depression in pediatric patients with FAP and functional nausea [3], stressor-induced gastric acid secretion and direct central nervous system initiation of nausea may also be contributing to symptoms.

### 2.3. Dietary/Microbiome Etiology

As with other functional gastrointestinal disorders, dietary composition and the intestinal microbiome are likely to be key players in the evolution and course of chronic functional nausea as well [23,24]. The influence of diet and the microbiome, however, have yet to be studied for this condition.

## 3. Diagnosing and Defining Functional Nausea

In the absence of “red flag” symptoms that prompt further evaluation, functional nausea is a clinical diagnosis that does not require additional testing, with the possible exception of gastric emptying studies [6].

Although neither measures chronicity, there are currently two validated tools for measuring pediatric nausea that are not specific to post-operative or chemotherapy-induced nausea. The first is the Nausea Profile, which is comprised of three nausea dimensions: (1) gastrointestinal (GI) distress; (2) emotional distress; and (3) somatic distress [11,25]. The Nausea Profile is a self-report symptom checklist designed to evaluate the experience of nausea and assess how it changes for an individual during or following an intervention. The second validated pediatric measure is the Baxter Retching Faces Scale (the BARF scale) [26]. The BARF scale is a child appropriate pictorial scale to assess nausea severity; however, it has only been validated for acute nausea. Unfortunately, these scales have limited applicability for evaluating functional nausea, as neither assesses chronicity or quality of life. Future research needs to address the development of diagnostic criteria and tools for evaluation of pediatric functional nausea. This will enable researchers and clinicians to better define and assess treatment response in this understudied patient population.

Given the high prevalence of nausea in the FAP pediatric population and its correlation with markers of physical and psychological health, future revisions of the Pediatric Rome diagnostic guidelines should consider defining functional nausea as a distinct entity.

## 4. Co-Morbidities

Pediatric patients with nausea and functional abdominal pain have more severe gastrointestinal and somatic symptoms, as well as poorer psychosocial functioning, than those with abdominal pain not complicated by nausea [3]. These differences remain significant after controlling for baseline severity of abdominal pain, suggesting that chronic nausea is an independent risk factor for worse health outcomes in children. Three pediatric studies to date have found associations between chronic nausea and poor health outcomes in children [1,2,3]. Levy *et al*. studied 132 children, ages 7–17 years, with a medical diagnosis of FAP [27]. Children who reported “high nausea” (*n* = 77), compared to children with “low nausea”, reported greater functional disability, more missed school days, and higher levels of anxiety and depression. Kovacic *et al.* performed a similar study in 2013 of over two-hundred pediatric patients, ages 6–18 years, with chronic abdominal pain [1]. Fifty-three percent of those reporting nausea had nausea at least twice a week and 28% experienced nausea daily. Nausea frequency was significantly correlated with poorer school and social functioning in this study and predicted social disability beyond pain. In this cohort, nausea was associated with headache, early satiety, fatigue, fullness, and heartburn. The most extensive study of nausea in pediatric patients to date was performed by our group at Monroe Carrel Jr. Children’s Hospital at Vanderbilt University [3]. Participants were enrolled in an institutional review board (IRB) approved prospective study of FAP in children ages 8–17 years. The sample included 871 FAP patients seen in a tertiary pediatric gastroenterology practice. Forty-five percent of children reported significant nausea within the preceding two weeks. These children, compared to FAP patients without nausea, reported significantly worse abdominal pain, gastrointestinal and somatic symptoms, depression, and functional disability. All of these differences remained significant when controlling for abdominal pain severity.

## 5. Treatment Strategies

There is no standardized treatment for children with chronic functional nausea. Several medications, complementary medicine techniques, and endoscopic interventions have promising preliminary data but need more rigorous evaluation before they can be considered standard of care. Interventions with supporting pediatric data are summarized in Table 1.

### 5.1. Pharmacotherapy

Odansetron: While odansetron, a 5-HT_3_ receptor antagonist, is frequently prescribed for acute and post-operative nausea or nausea secondary to chemotherapy, there is a scarcity of evidence for its use for functional nausea. All data thus far are from small adult studies. A placebo-controlled adult study of 22 patients with post-infectious functional gastrointestinal disorders showed improvement in postprandial nausea with odansetron administration, but no change in gastric hypersensitivity or gastric emptying [27]. There is not enough data to make a recommendation for its use in pediatric chronic nausea at this time.

Fludrocortisone: Sixteen female adolescents with chronic unexplained nausea and orthostatic intolerance were given 0.1–0.2 mg/day of the aldosterone agonist fludrocortisone for a minimum of four weeks and all had significant improvement in their nausea [12]. Treatment also resulted in improved abdominal pain, dizziness, flushing, and school attendance. Based on this study, a trial of fludrocortisone in patients with nausea with documented orthostatic intolerance may be beneficial. Assessment of orthostatic symptoms during the gastroenterology clinical encounter is necessary, however, to determine which patients may benefit from this medication. A basic screen would be assessing heart rate following an abrupt position change from supine to upright, with an increase in 40–45 beats per minute being suggestive of postural orthostatic tachycardia syndrome [20,28,29].

Cyproheptadine: Cyproheptadine is an antagonist of serotonin, histamine H1, and muscarinic receptors. It has been shown to be safe and effective for treating dyspeptic symptoms in children, including those with nausea, possibly by increasing gastric accommodation [30]. In 24 children with nausea associated with dyspepsia, 50% had significant improvement or resolution of symptoms at a median dose of 6 mg/day of cyproheptadine over a median 20-week treatment course [30]. However, one third of patients in this retrospective, open-label study reported side effects from the cyproheptadine. These included somnolence, behavioral changes, increased appetite and weight gain, and abdominal pain. The majority of these side effects resolved with time or dose reduction. Prospective, placebo controlled studies are needed.

Amitriptyline: Retrospective pediatric data has shown a 50% improvement in nausea symptoms with the tricyclic antidepressant amitriptyline at a mean dose of 50 mg nightly [31]. Amitriptyline may be effective for visceral analgesia via facilitation of GABA-ergic neurotransmission, blocking both serotonin and norepinephrine reuptake, and possibly potentiating the endogenous opioid system. Tricyclic antidepressants, however, can have multiple undesirable adverse effects, with anticholinergic effects being the most pronounced with amitriptyline specifically.

### 5.2. Phytotherapy

Ginger: Ginger (*Zingiber officinale*) has been traditionally used to treat indigestion, nausea, and vomiting. Ginger extracts improve gastric emptying and stimulate gastric antral contractions [32]. A randomized, double-blind, placebo controlled study of 126 adults with functional dyspepsia was recently performed using an extract of ginger and artichoke (Cynara cardunculus) [33]. After 14 days of treatment, the supplementation group had significant improvement in nausea, epigastric pain and fullness, and bloating compared to the placebo group with no adverse events reported. In regards to inhalational use, there is evidence that ginger essential oil aromatherapy reduces the incidence and severity of nausea, decreases antiemetic requirements, and improves patient satisfaction, although studies have been limited to those with post-operative or chemotherapy-related nausea [32]. Pediatric data is needed.

Peppermint: Peppermint oil (*Mentha piperita)* has been shown to be effective for post-operative and pregnancy related nausea [32]; less is known regarding functional nausea. An herbal preparation containing extracts of peppermint, caraway fruit, fennel, and wormwood, has been shown to significantly reduce nausea in adults with functional upper abdominal complaints and was significantly better tolerated than metoclopramide [34]. Given that peppermint has already been proven safe and effective for pediatric irritable bowel syndrome [35], its use for functional nausea warrants further investigation. One concern is that it may aggravate or induce dyspepsia, and so the best mode of administration must be evaluated for those with nausea.

### 5.3. Mind-Body Techniques

Hypnotherapy: Hypnotherapy has been evaluated in a randomized controlled trial for children with functional gastrointestinal disorders. Six 50-min sessions over three months significantly decreased associated symptoms, including nausea, more than standard medical therapy [36]. Additionally, hypnotherapy has been successfully used for anticipatory nausea in children [37,38]. This may be a useful adjunct in the treatment of functional nausea.

Biofeedback: Biofeedback is a low risk intervention to help pediatric patients restore autonomic balance by improving sympathovagal tone and has shown promise in children with functional abdominal pain [39]. Biofeedback will need to be evaluated in the chronic functional nausea population before formal recommendations can be made, but is a low risk suggestion for interested families.

Acupuncture/Acupressure: Acupuncture, electro-acupuncture and acupressure of the PC-6 point (located on the forearm 3–5 cm from the flexor crease) can be beneficial for post-operative, pregnancy, and chemotherapy-induced nausea [40]. Acupuncture has shown promise for the treatment of adult irritable bowel syndrome in several randomized controlled trials [41], but there are no data at this time to support or refute its use for pediatric functional disorders.

Mental Health Support: Given the high incidence of co-morbid depression, anxiety, and functional disability reported in children with functional nausea, as well as the increased risk for generalized anxiety and major depressive episodes in adulthood [3], an integrative approach that addresses mental health and coping strategies is vital to treatment success. It is our opinion that all pediatric patients with functional nausea should be screened for anxiety and depression and offered referral for mental health support and treatment as needed.

### 5.4. Gastric Electrical Stimulation

Gastric electrical stimulation (GES), also known as a gastric pacemaker, has been used for adults with dyspepsia, gastroparesis, and severe nausea and vomiting without gastroparesis that is refractory to medical and dietary management. It consists of an implanted neurostimulator that administers high frequency, low-energy electrical stimulation to the antrum of the stomach via implanted electrodes. Experience and data on GES in pediatric patients is limited but growing [42,43,44]. In the largest series of twenty-four adolescent patients with functional dyspepsia, 60% of which had gastroparesis, there were statistically significant improvements in multiple areas of quality of life, most notably in nausea severity, following GES [42]. The average frequency of nausea prior to gastric electrical stimulation was greater than seven times per week, but following treatment the average frequency was rare or once weekly. This improvement was seen both in children with delayed and normal gastric emptying. GES has been used successfully in children as young as two years of age, and should be considered for refractory cases of nausea [43].

## 6. Conclusions

Chronic functional nausea needs to receive more attention as a pediatric FGID and may warrant definition as a distinct disorder in the next iteration of the pediatric Rome Criteria. This will allow for better classification and representation of this population in research studies to better understand its etiology and further the development of effective interventions. The pathophysiology of this disorder remains unclear, but likely involves a complex interplay of autonomic, psychosocial, dietary, microbial, and gastric sensorimotor disturbances. Cyproheptadine and tricyclic antidepressants are the mainstays of medical treatment, with fludrocortisone offering potential benefit to those with orthostatic induced symptoms. Traditional medical management still only offers at most a 50% improvement in symptoms and does not address the psychological comorbidities of functional nausea. An integrative medicine approach may offer additional benefit, but further studies are needed before specific recommendations can be made.

## Figures and Tables

**Table 1:** Summary of treatments for pediatric chronic functional nausea with pediatric evidence.

Intervention	Dose	Mechanism of Action	Precautions
Fludrocortisone [12]	0.05–0.2 mg/day	Treatment of gastric dysrhythmias secondary to orthostatic intolerance	Contraindicated with systemic fungal infections
Cyproheptadine [30]	0.04–0.6 mg/kg/day divided BID or TID (9 months and older)	Serotonin, histamine, and muscarinic receptor antagonist	Side effects: somnolence, behavioral changes, weight gain
Amitriptyline [31]	30–50 kg: 10 mg at bedtime 50–80 kg: 20 mg at bedtime >80 kg: 30 mg at bedtime [45] Titrate as needed to maximum dose of 200 mg divided BID for adolescents.	Facilitation of GABA-ergic neurotransmission, possible potentiation of endogenous opioid system	Pre-screen for idiopathic long QT syndrome
Hypnotherapy [36,37,38]	Twice monthly 50 min sessions × 3 months	Possible influence on the brain-gut axis	Contraindicated in patients with psychosis
Gastric Electrical Stimulation [42,43,44]	Implanted neurostimulator in the stomach antrum; age 2 years and older	High frequency, low energy electrical stimulation of the stomach	Discomfort at implantation site and battery failure can occur

BID: twice a day; TID: three times a day

## Abbreviations

The following abbreviations are used in this manuscript:

FAP	functional abdominal pain
FGID	functional gastrointestinal disorders
CIN	chronic idiopathic nausea
GES	gastric electrical stimulation
